# Activation of Fms-Like Tyrosine Kinase 3 Signaling Enhances Survivin Expression in a Mouse Model of Rheumatoid Arthritis

**DOI:** 10.1371/journal.pone.0047668

**Published:** 2012-10-17

**Authors:** Sofia E. M. Andersson, Mattias N. D. Svensson, Malin C. Erlandsson, Mats Dehlin, Karin M. E. Andersson, Maria I. Bokarewa

**Affiliations:** Department of Rheumatology and Inflammation Research, Sahlgrenska University Hospital, University of Göteborg, Göteborg, Sweden; University of Lyon, France

## Abstract

Survivin is known as an inhibitor of apoptosis and a positive regulator of cell division. We have recently identified survivin as a predictor of joint destruction in patients with rheumatoid arthritis (RA). Flt3 ligand (Flt3L) is expressed in the inflamed joints and has adjuvant properties in arthritis. Studies on 90 RA patients (median age 60.5 years [range, 24–87], disease duration 10.5 years [range, 0–35]) show a strong positive association between the levels of survivin and Flt3L in blood. Here, we present experimental evidence connecting survivin and Flt3L signaling. Treatment of BALB/c mice with Flt3L led to an increase of survivin in the bone marrow and in splenic dendritic cells. Flt3L changed the profile of survivin splice variants, increasing transcription of the short survivin40 in the bone marrow. Treatment with an Flt3 inhibitor reduced total survivin expression in bone marrow and in the dendritic cell population in spleen. Inhibition of survivin transcription in mice, by shRNA lentiviral constructs, reduced the gene expression of Flt3L. We conclude that expression of survivin is a downstream event of Flt3 signaling, which serves as an essential mechanism supporting survival of leukocytes during their differentiation, and maturation of dendritic cells, in RA.

## Introduction

Survivin is an intracellular protein and a member of the inhibitor of apoptosis proteins (IAPs), with many functions in cytoprotection, cell division and cellular adaptation. It is encoded by the *BIRC5* gene on the human chromosome 17q25, into a 142 amino acid, 16.5 kDa protein but can be extensively alternatively spliced into several variants and form homodimers and heterodimers with two different splice variants, or an IAP-IAP complex by pairing with X-linked IAP (XIAP) [1], [2]. Transcription of survivin is negatively regulated by p53 [3] and positively regulated by STAT3 [4] and TCF-4 [5].

Similar to human protein, full-length murine survivin (survivin140) contains a single BIR-domain, which is critical for its anti-apoptotic function [6], and a carboxy-terminal coiled-coil domain that links its function to the cell cycle [7]. Murine survivin121 contains a BIR-domain that makes it able to inhibit caspase activity but lacks the coiled-coil structure. Additionally, there is a splice variant that predicts a 40 aa residue protein (survivin40), which lacks both the BIR- and a coiled-coil regions. The differential expression of these forms of survivin is believed to affect the balance between cell proliferation and programmed cell death [8].

Survivin has only limited expression in adult tissues, but is overexpressed in tumors and is therefore regarded as a cancer gene. Survivin is expressed in a cell-cycle dependent manner with a peak at G2/M. Together with Aurora B, borealin and INCENP, survivin forms the chromosomal passenger complex, which is recruited to chromosomes by the phosphorylation by histones which is recognized by the BIR domain of survivin. In anaphase, the chromosomal passenger complex relocalizes to form the mitotic spindle and stimulate cytokinesis. The requirement of survivin during fetal development has been demonstrated by lethality of knockout embryos [1].

Survivin is also expressed independently of cell cycle progression and is linked to the inhibition of apoptosis [3]. The pool of survivin with apoptotic inhibiting properties seems to be localized to the mitochondria [1], [9], and is released into the cytoplasm in response to death stimuli. Survivin inhibits apoptosis in complex with hepatitis B X interacting protein [10] or XIAP, perhaps by separating Smac/Diablo from XIAP, thus enabling caspase degradation [1]. There is an emerging role for survivin in normal adult CD34+ hematopoetic stem cells [11], [12] and in the development, maturation and survival of immune cells, for example in T cells [13]–[16] and neutrophils [17]. It is upregulated in response to stimulation with hematopoetic cytokines and growth factors [18]–[20].

Expression of survivin in malignancies is associated with unfavorable outcome [21], [22] and resistance to cytotoxic treatment [23]–[25]. In the context of rheumatoid arthritis (RA), extracellular survivin is a marker of poor prognosis. A prospective study on 651 patients at the early stage of RA with the mean disease duration of 6 month showed that high serum levels of survivin were predictive for severe cause of RA, characterized by persistent joint inflammation and progressive joint destructions [26]. The proportion of survivin-positive patients may vary from 20–30% in the group of established and treated RA patients [27] to 60% in the population of early RA patients [26]. Successful anti-rheumatic treatment may reduce serum levels of survivin, while survivin-positive patients accumulate among the patients who do not respond to anti-rheumatic treatment [26]–[28]. A growing number of publications [26], [27], [29]–[31] support the idea that survivin has a role in progression of RA. We have previously shown that survivin has a key function in the regulation of invasive properties of fibroblasts in the inflamed rheumatic joint, and that intracellular survivin is essential for urokinase expression and for the up-regulation of urokinase receptor [32]. Also, serum survivin modifies surface pattern of leukocyte adhesion molecules [33].

Fms-like tyrosine kinase 3 (Flt3) is a receptor tyrosine kinase class III, a class that also includes c-KIT, PDGFRα/β and c-FMS receptors. It is expressed on hematopoietic stem cells and promotes differentiation, maturation and survival of lymphoid progenitors in the bone marrow [34]. Flt3 is essential for the development of antigen presenting cells, such as B cells and dendritic cells (DC) [35]–[37]. Mutations in the Flt3 receptor, leading to its constant activation, are frequently found in acute myeloid leukemia (AML) [38]. It has been shown that bone marrow cells expressing Flt3 with internal tandem duplication mutations which keeps Flt3 signaling constantly active, have an increased survivin expression [39], [40] and this is associated with development of resistance to Flt3 inhibition [39].

Flt3 ligand (Flt3L) is expressed by many cell types, and is present in a soluble intracellular form, and in a membrane bound form [41]. Flt3L has recently been outlined within a panel of preclinical biomarkers of predictive value for the development of RA [42]. We have recently shown that Flt3L is elevated in RA patients and that Flt3L has adjuvant properties when injected into the joint, facilitating development of arthritis in experimental settings [43]. Blockade of Flt3 signaling using a synthetic Flt3 inhibitor alleviates signs of synovitis and cartilage destruction in antigen-induced model of arthritis [44].

Since expression of the Flt3 receptor and survivin in adults is present in hematopoietic stem cells, we address the question whether Flt3L signaling could actually be linked to survivin in rheumatoid arthritis. We further tested the pathological significance of this relation using in vivo treatment with Flt3L and evaluated the possibility of targeting this pathway with an Flt3 inhibitor and lentiviral shRNA knockdown of survivin.

## Results

### Higher Levels of Flt3 Ligand in Survivin Positive Rheumatoid Arthritis Patients

**Figure 1:**
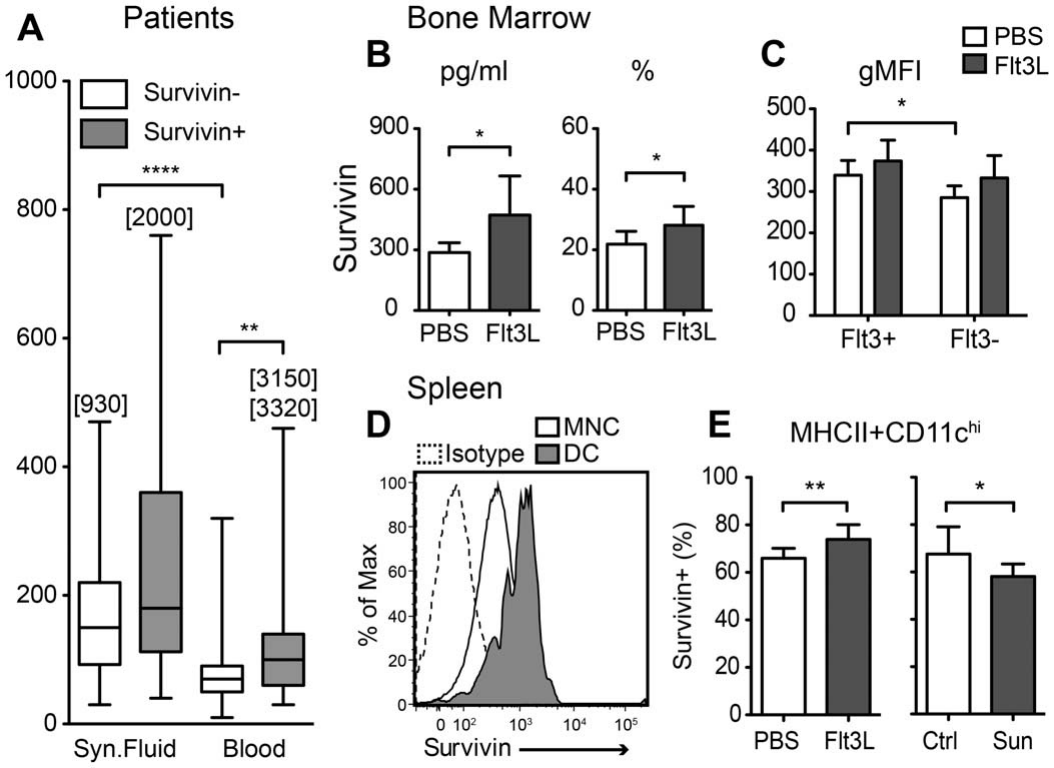
Treatment with Flt3 ligand increases expression of surviving. (A) Levels of Flt3L in blood and synovial fluid of patients with rheumatoid arthritis. Stratification of patients was done into the groups of survivin+ (serum or synovial fluid levels above 450 pg/ml, n = 29) and compared to survivin- (serum and synovial fluid levels below 450 pg/ml, n = 61). (B) Survivin levels in bone marrow from Flt3L or PBS treated mice measured by sandwich ELISA (left panel; Flt3L, n = 8; PBS, n = 8), and the percentage of the survivin positive bone marrow cells measured by flow cytometry (right panel; Flt3L, n = 7; PBS, n = 11). (C) Flt3L treatment increased the intensity of survivin (expressed as gMFI) in the Flt3+ and Flt3- cell populations. (D) Flow cytometry histogram showing survivin staining in spleen MHCII+CD11c^hi^ dendritic cells (dark) compared to total spleen mononuclear cells (open) and isotype control staining (dashed). (E) Percentage of survivin positive cells in the MHCII^+^CD11c^hi^ dendritic cell population in the spleen after treatment with Flt3L (n = 7) or Flt3-inhibitor sunitinib (Sun) (n = 10) compared to control groups (n = 10 and 11 respectively). Comparison between the groups was done by the Mann-Whitney statistics. (*), p<0.05; (**), p<0.01; (***), p<0.001.

Survivin levels in the blood of 104 healthy controls (male n = 16, female n = 88, mean age 52.4 years [range 18–67]) were measured by ELISA. The evaluation of the results showed a non-Gaussian distribution of survivin levels in the studied group. The 95% confidence interval was calculated which indicated that the levels of survivin above 450 pg/ml was present in less than 5% of healthy individuals. Thus, the patients with serum or synovial fluid levels of survivin above 450 pg/ml represented the survivin-positive group (n = 29) and the remaining were survivin-negative (n = 61, Table 1). In the present material, 32% of the RA patients were survivin-positive. This is in agreement with our previous reports [27]. As expected, survivin-positive RA patients were significantly more often RF-positive (p = 0.0006) and a majority of the survivin positive patients had erosive joint disease (p = 0.003). The comparison between the groups revealed significantly higher levels of Flt3L in the blood of the survivin-positive RA patients compared to survivin-negative (pg/ml: 110 [range 30–3320] vs 70 [range 10–230], p = 0.003, Fig. 1A). This was also the case for synovial fluid (pg/ml: 200 [range 40–2000] vs 150 [range 30–930], p = 0.055), although not statistically significant (Fig. 1A). There was no significant difference in the levels of acute-phase reactants (e.g., C-reactive protein, serum amyloid A protein levels, IL-6 levels) between the survivin-positive and survivin-negative groups (Table 1).

**Table 1:** Clinical characteristics of patients with rheumatoid arthritis.

	Survivin+*n* = 29	Survivin−*n* = 61	p-value*
Gender, f/m (n)	22/7	45/16	n.s.
Age, years	60 [24–84]	61 [28–84]	n.s.
Rheumatoid factor + (n, %)	25 (86%)	31 (41%)	0.0006
Disease duration, years	8 [1]–[33]	9 [1]–[35]	n.s.
Erosions (n, %)	25 (86%)	35 (57%)	0.003
Treated with DMARDs** (n, %)	22 (76%)	35 (57%)	n.s.
Methotrexate (n, %)	19 (66%)	26 (43%)	n.s.
Other (n, %)	3 (10%)	9 (15%)	n.s.
Non-treated (n, %)	7 (24%)	26 (43%)	n.s.
CRP, mg/L	29 [5–230]	28 [5–170]	n.s.
SAA, mg/L	100 [22–600]	42 [22–600]	n.s.
IL-6, pg/ml (SF)	1.68 [0.03–4.52]	1.81 [0.05–2.69]	n.s.
WBC count,×10^9^/L			
blood	8.0 [3.6–16.8]	8.2 [5.5–14.2]	n.s.
synovial fluid	15.2 [0.1–43.8]	10.4 [0.7–33.2]	n.s.
Survivin, ng/ml			
blood	2.5 [0.45–160]	0 [0–0.38]	<0.0001
synovial fluid	1.81 [0.53–40]	0 [0–0.37]	<0.0001

Data presented as median [range].

* *p*-values are referred to a comparison between the groups of Survivin+ and survivin- patients, calculated using Mann-Whitney U test or Chi-Square test for categorial data.

** DMARDs, disease modifiying anti-rheumatic drugs SAA, Serum Amyloid A protein. SF, synovial fluid. CRP, C-reactive protein. n.s  =  not significant.

### Survivin Expression is Upregulated in Mouse Bone Marrow Following Treatment with Flt3 Ligand

To elucidate a causative relation behind the observed association between survivin and Flt3L levels in RA patients, arthritic mice were challenged with Flt3L. Two independent experiments were performed containing 15 Flt3L-treated mice and 19 PBS-treated controls. After 14 days of Flt3L treatment, survivin levels in bone marrow cell lysates were increased compared to the PBS treated group (pg/ml: 472.5±193.4 vs 286.5±48.9, *p* = 0.01, Fig. 1B). At day 28, the number of survivin+ cells in bone marrow of Flt3L treated mice was higher compared to PBS treated controls (28.1±6.3% vs 21.9±4.3%, *p* = 0.02, Fig. 1B). Additionally, Flt3L-treated mice tended to have somewhat higher survivin concentration per cell, measured as the intensity of intracellular staining (gMFI: 336±54 vs 288±29, n.s.). The intensity of survivin staining was higher in the Flt3+ cells compared to the Flt3− cell population (*p* = 0.003). Flt3L treatment tended to upregulate survivin expression in both Flt3+ and Flt3− cell populations (Fig. 1C).

**Figure 2:**
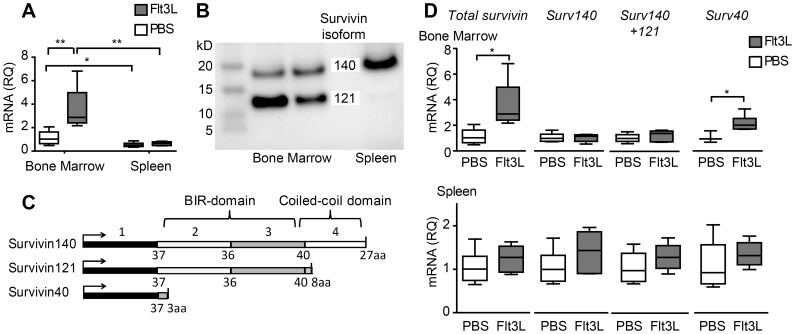
The effect of Flt3L-treatment of BALB/c mice on the expression profile of the survivin splice variants. Mice treated with Flt3L (n = 6) or PBS (n = 6) for 14 days. (A) Total survivin gene expression in bone marrow and spleen, RQ  =  relative quantity, all values are related to bone marrow from PBS-treated mice. (B) Western blot showing survivin protein isoforms survivin140 and survivin121 in cell lysates from bone marrow and spleen. (C) A schematic overview of the three splice variants of survivin in mice. (D) Gene expression of the different survivin transcripts in bone marrow and spleen, RQ  =  relative quantity, Flt3L-treated related to PBS-treated for each assay. Data shown as median, whiskers  =  min to max, (*) p<0.05, (**) p<0.01.

Despite the similar size of survivin+ population in bone marrow and spleen, survivin gene expression in spleen was 4 times lower compared to bone marrow (p = 0.002, Fig. 2A, PBS). Flt3L treatment of arthritic mice did not affect total survivin levels in the spleen, since the percentage of survivin+ spleen cells was similar in the Flt3L treated group and the control group (20.3±5.3% vs. 19.5±6.3%). High intensity of survivin expression was attributed to the MHCII^+^CD11c^hi^ dendritic cell population in the spleen (Fig. 1D). Flt3L treatment increased the proportion of survivin+ cells within the MHCII^+^CD11c^hi^ population (73.9±6.2% vs 66.0±4.2%, p = 0.005, Fig. 1E). When cultured *in vitro*, splenocytes of Flt3L-treated mice (n = 7) secreted significantly higher levels of survivin compared to splenocytes of PBS-treated controls (n = 7) (pg/ml: 32.6±7.7 vs 24.3±6.2, p = 0.04), but Flt3L treatment did not increase serum levels of survivin (pg/ml: 1772±2503 vs 1190±1335).

### Flt3L Changes the Profile of Survivin Splice Variants in Bone Marrow

Gene expression analysis showed an increase in the overall transcription of *survivin* in the bone marrow of Flt3L-treated mice (*n* = 6) compared to PBS treated controls (*n* = 6) (Fig. 2A). This increase was not seen in spleen. Moreover, *survivin* gene expression in spleen was lower compared to the expression in the bone marrow (*p* = 0.002, Fig. 2A). Further analysis of different splice variants of survivin (Fig. 2C) was done on spleen and bone marrow samples. Flt3L treatment increased transcription of the short *survivin40* variant in bone marrow, while no increase in the transcription of the full-length variant *survivin140* could be detected (Fig. 2D). Since it was hard to design specific primers for the *survivin121* transcript, a primer pair localized in the exon 1 and exon 2 was used. These primers were able to detect *survivin140* and *survivin121* transcripts, but not *survivin40* transcript. The transcription of *survivin140* and *survivin121* variants was similar in Flt3L treated and in PBS treated mice. Western blot showed the dominating expression of survivin140 in spleen and survivin140 and survivin121 in bone marrow samples (Fig. 2B).

### Survivin Expression in the Bone Marrow is Dependent on Flt3 Receptor Activity

**Figure 3:**
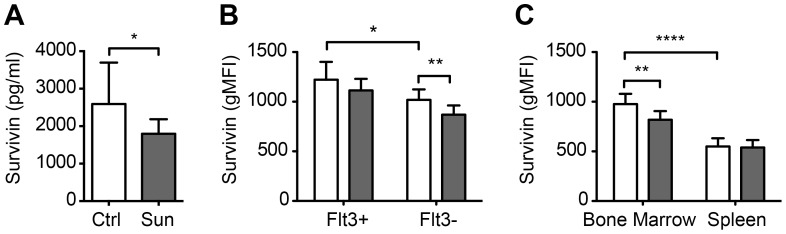
Survivin levels in bone marrow and spleen of BALB/c AIA mice after Flt3 inhibition in vivo. Survivin levels in bone marrow and spleen of mice treated with Flt3 inhibitor sunitinib (Sun, 40 mg/kg/d, n = 10, grey bars) compared to control mice treated with citric acid (Ctrl, *n* = 10, open bars). (A) Survivin levels in bone marrow lysates, measured by sandwich ELISA. (B) The intensity of survivin (expressed as gMFI) in bone marrow Flt3+ and Flt3- cells, and (C) in total bone marrow and spleen. This experiment was repeated with similar results. Comparison between the groups was done by the Mann-Whitney statistics. (*), p<0.05; (**), p<0.01; (***), p<0.001.

We hypothesized that blockade of Flt3 receptor would affect survivin production in the bone marrow. Survivin was measured in arthritic mice treated with the Flt3 inhibitor SU11248 (sunitinib) in 3 independent experiments containing totally 24 sunitinib-treated mice and 25 control mice treated with citric acid. On day 28, a reduction of total survivin levels was observed in bone marrow lysates of sunitinib treated mice compared to the citric acid treated controls (pg/ml: 1796±388 vs. 2590±1107, p = 0.049, Fig. 3A). Sunitinib significantly reduced the number of survivin+ cells in mouse bone marrow (46.8±4.2% vs 59.3±6.8%, p = 0.0007), and the intensity of intracellular survivin staining (p = 0.002, Fig. 3C). This reduction of survivin expression could be seen mainly on Flt3− cells (*p* = 0.003, Fig. 3B).

To assess whether sunitinib suppressed the expression of survivin in secondary lymphoid organs, we analyzed survivin levels in the spleen. The total survivin levels in the spleen were not reduced following sunitinib treatment (Fig. 3C), however a significant reduction of survivin expression in the MHCII^+^CD11c^hi^ dendritic cell population was identified (gMFI: 2684±607 vs 2179±207, p = 0.02). The number of survivin+ dendritic cells was also reduced with sunitinib treatment (58.1±5.3 vs 67.6±11.5%, p = 0.05, Fig. 1E). In spleen, Flt3 inhibition resulted in a reduction of CD11c+ cell population (p = 0.02, Table 2). The latter observation was in consistence with our previous findings on the inhibitory effect of sunitinib on the development of dendritic cells [44].

**Table 2:** Percentage of major leukocyte populations in bone marrow and spleen of sunitinib treated mice.

	Bone Marrow			Spleen		
	Control	Sunitinib	P value	Control	Sunitinib	P value
CD3+ (%)	8.7±0.8	7.3±0.7	0.001	30.2±1.5	31.8±4.7	n.s.
B220+ (%)	26.2±4.6	22.0±4.0	0.04	56.0±7.1	54.0±5.7	n.s.
CD11b+ (%)	49.0±4.3	59.5±6.7	0.002	4.2±0.9	3.9±1.2	n.s.
Gr-1 (%)	47.0±4.3	55.1±6.2	0.007	12.9±1.2	13.4±1.4	n.s.
CD11c+ (%)	2.9±0.3	3.1±0.7	n.s.	4.1±1.5	2.8±0.7	0.02

1–3 pooled experiments, 9–19 mice, n.s  =  not significant.

### Survivin shRNA Reduces Flt3 Ligand Expression in the Spleen

**Figure 4:**
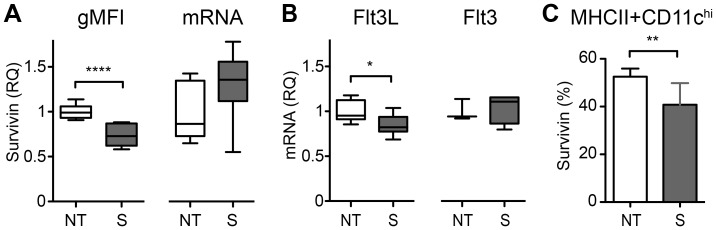
*In vivo* downregulation of survivin using shRNA in BALB/c mice. Lentiviral particles containing a sequence coding for shRNA targeting survivin gene (S, n = 8) were injected intra-articular. The control group received lentiviral particles containing non-targeting shRNA (NT, n = 9). (A) Levels of survivin protein (survivin gMFI analyzed using flow cytometry) and survivin mRNA (RT-qPCR analysis of survivin gene expression) in the spleen (NT, n = 9; S, n = 8). (B) *Flt3L* (NT, n = 7; S, n = 7) and *Flt3* (NT, n = 3; S, n = 4) gene expression in spleen tissue. (C) Percentage of survivin positive MHCII^+^CD11c^hi^ dendritic cells in the spleen analyzed using flow cytometry (NT, n = 9; S, n = 8). Comparison between the groups was done by the Mann-Whitney statistics. (*), p<0.05; (**), p<0.01; (***), p<0.001.

Finally, we studied if Flt3/Flt3L system was changed by survivin inhibition. BALB/c mice received lentiviral constructs, containing a sequence coding for shRNA targeting survivin gene transcripts (n = 8), or the same lentiviral construct containing a non-targeting shRNA sequence (n = 9), by a single intra-articular injection. On day 28, the efficacy of survivin inhibition was confirmed on the transcription level by qPCR and on the protein level by flow cytometry. shRNA successfully reduced the total population of survivin+ cells by 35% (17.1±5.2 vs 26.4±3.4, *p* = 0.002), and the intensity of survivin expression in the spleen (gMFI: 582±95 vs 789±61, p<0.0001, Fig. 4A). The gene transcription of survivin in spleen tissue and bone marrow was not significantly changed (Fig. 4A and data not shown). Inhibition of survivin led to a reduction in Flt3L transcription in the spleen (0.85±0.12 vs 1.01±0.12, *p* = 0.04, Fig. 4B), and to a reduction of survivin+ MHCII^+^CD11c^hi^ dendritic cells (40.8±9.1 vs 52.5±3.4, *p* = 0.006, Fig. 4C).

## Discussion

High levels of survivin and Flt3L in blood and synovial fluid of patients with rheumatoid arthritis are implied in the pathogenesis of joint inflammation [26], [27], [43]. Additionally, a tight clinical association observed between these parameters with respect to joint destruction and effect of anti-rheumatic treatment [28] prompted us to study common biological processes linking high expression of survivin and Flt3L in RA patients. The analysis of a cohort of RA patients with the established joint disease showed a clear association of high Flt3L levels with high levels of survivin in blood. Neither Flt3L nor survivin was a consequence of inflammation, measured by CRP, WBC count, serum amyloid A protein and IL6. Due to the cross-sectional nature of the study, one can see a clear difference in the frequency of radiologic erosions, which is a sign of severe disease, in the survivin positive group compared to the survivin negative group. The survivin-positive group contains higher proportion of patients treated with immunosuppressive anti-rheumatic drugs compared to the survivin-negative group. High levels of survivin characterize patients with therapy resistant cause of RA, suggesting that many patients responding to anti-rheumatic treatment convert from survivin-positive to survivin-negative. The results from the patient material show an association between Flt3L and survivin in RA, suggesting the existence of a mutual mechanism regulating levels of these proteins. Similarity of the groups with respect to disease duration, age, levels of C-reactive protein and white blood cell count further support this argument.

Our clinical observations were further extended in the experimental setting, where the requirement of Flt3/Flt3L signaling for expression of survivin *in vivo* was assessed. Survivin levels were increased following treatment with recombinant Flt3L and they were decreased with Flt3 receptor blockade. This proves that survivin production in the bone marrow is dependent on Flt3 signaling and makes Flt3L a major trigger of survivin transcription and expression in leukocytes. Interestingly, we showed the existence of a negative regulation loop where the low expression of survivin in spleen (induced by shRNA) resulted in the low levels of Flt3L in spleen. In the joints of RA patients, the elevated levels of Flt3L could originate from immune cells or fibroblast-like stromal cells in the synovium, by an inflammation caused shedding of the membrane bound form [45] or as a response to lack of antigen presenting cells. It is for example known that the human syndrome of dendritic cell, monocyte, B and NK lymphoid deficiency is associated with elevated levels of Flt3L in humans [37] and the same is true in mice following Flt3 inhibition [44], [46].

Flt3/Flt3L signaling is important for the maturation of hematopoietic cells [34]. Here, we show that Flt3L induces survivin production in the Flt3+ progenitors in bone marrow, and in dendritic MHCII^+^CD11c^hi^ cells in spleen. Survivin is increased after Flt3L treatment also in the Flt3− population in the bone marrow, suggesting that survivin is induced in Flt3 positive hematopoietic progenitors and remains upregulated after internalization of activated Flt3. Thus, survivin expression induced by Flt3L is associated with differentiation of cells rather than cell proliferation. Our findings are in agreement with previous reports on the induction of survivin production in CD34+ stem cells [20] prior to their differentiation.

Spleen is a maturation site for the peripheral dendritic cells and the process has recently been shown to be dependent on Flt3 expression [47], [48]. In our study, a reduction of the intracellular pool of survivin by inhibition of Flt3 led to a pronounced reduction of CD11c+ population of dendritic cells in the spleen. The obvious effect of Flt3L treatment/Flt3 inhibition on survivin levels in the MHCII^+^CD11c^hi^ population in the spleen further supports a strong relationship between the induction of survivin and DC development. We have previously shown that inhibition of Flt3 receptor reduces the DC population in spleen [44]. In RA patients, we have seen that high levels of extracellular survivin are associated with an increased number of circulating CD11c+ survivin expressing cells in blood [33]. Our present results suggest that modulation of survivin levels is an essential mechanism regulating homeostasis of peripheral dendritic cells.

Interestingly, studies on the Flt3L-induced expressions of different survivin-transcripts revealed an effect on the short *survivin40*-transcript in bone marrow. This variant lacks both the BIR-domain, essential for the interaction with caspases and for the anti-apoptotic effects of survivin [6], as well as the coiled coil-domain required for the formation of chromosomal passenger complex [7]. As survivin40 retains the N-terminal domain, it has been believed to bind other survivin variants [8], and to modulate their function by preventing homodimerization and facilitating interaction through the BIR- and coiled-coil domains. However, we could not detect survivin40 on protein level with western blot, as this has been shown earlier by another group [8] and might either indicate that this transcript is not translated or that the antibodies used are not able to detect the survivin40. The increase in survivin protein after Flt3L treatment can either be a result of an increased survivin40-expression or of a decrease in survivin140- or survivin121- turnover. In spleen, high survivin expression is seen within the MHCII^+^CD11c^hi^ population. This population represents a small part of the total amount of spleen cells and verification of survivin changes requires cell sorting prior to the RNA analyses.

In conclusion, our findings indicate that expression of survivin is a downstream event of Flt3L signaling, which serves as an essential mechanism supporting survival of hematopoietic cells during their differentiation and maturation of antigen presenting cells in bone marrow and spleen during arthritis.

## Materials and Methods

### Ethics Statement

The study involving human participants (S441-01) was approved by the Ethics Committee of Sahlgrenska University Hospital. All studies were conducted in compliance with the Declaration of Helsinki, and all patients gave written informed consent to participate in the study. All animal experiments (Protocol Numbers: 176-2008, 328-2008) were approved by the Ethical Committee on Animal Experiments in Gothenburg.

### Patients

Synovial fluid and serum samples were obtained from 90 patients with RA attending the Rheumatology Clinic at Sahlgrenska University Hospital, Göteborg, Sweden, for acute joint effusion. All the patients met the diagnostic criteria for RA suggested by the American College of Rheumatology (ACR) [49]. The presence of bone erosions, defined as the loss of cortical definition at the joint, was recorded in proximal interphalangeal, metacarpophalangeal, carpus, wrist and metatarsophalangeal joints using recent radiographs of hands and feet. The presence of one erosion was sufficient to fulfill the requirement of an erosive disease. The presence of rheumatoid factor of any of the immunoglobulin isotypes was considered positive. Clinical characteristics of the patients are presented in Table 1. Blood from 104 healthy blood donors (male n = 16, female n = 88, mean age 52,4 years [range 18–67]) was used to calculate normal levels of survivin.

### Collection and Preparation of Samples

Synovial fluid was obtained by arthrocentesis, aseptically aspirated and transmitted into sodium citrate (0.129 mol/l; pH 7.4) containing tubes. In most cases synovial fluid was obtained from knee joints. Synovial fluid samples were aliquoted and frozen in −70°C immediately after joint injections. Blood samples were centrifuged at 1000 rpm and serum was aliquoted and stored at -70°C.

### Laboratory Measures of Disease Activity

Serum levels of C-reactive protein (CRP) were measured by standard nephelometry, with established normal range 0–5 mg/ml. Serum amyloid A protein (SAA) levels were detected by an ELISA (Biosource, Camarillo, CA). White blood cell (WBC) counts in blood and synovial fluid samples were obtained with a microcell counter F300 (Sysmex, Toa, Japan). Levels of IL-6 were measured as described previously [43], and rheumatoid factor (RF) were measured using standard laboratory techniques at Sahlgrenska University Hospital, Göteborg, Sweden.

### Antigen Induced Arthritis using Methylated Bovine Serum Albumin (mBSA)

Female Balb/c mice were purchased from the Charles River Laboratory (Germany) and housed at the animal facility at the Department of Rheumatology & Inflammation Research, under standard conditions of temperature and light, and fed laboratory chow and water ad libitum. Mice (10 weeks old) were immunized with subcutaneous injection of mBSA (200 µg/mouse, Sigma-Aldrich) dissolved in PBS and mixed with IFA (Sigma-Aldrich) at day 0, followed by booster with mBSA (100 µg/mouse) at the tail root at day 7. Arthritis was induced by a single intra-articular injection of mBSA (30 µg/mouse) in PBS given at day 21. Mice were sacrificed on day 10 or 28. Samples of serum, bone marrow and spleen were collected for further analysis.

### *In Vivo* Treatment with Flt3 Ligand and Flt3 Inhibitor

Arthritic mice were treated with a daily intraperitoneal injection of recombinant Flt3L, in the dose of 1.5µg/mouse, starting 4 days prior to first immunization. Flt3L was purchased from Creative BioMart (Shirley, NY) or purified from Sp2.0 hybridoma transfected with Flt3L gene [43]. Control mice were treated with PBS injection or a control cell line supernatant purified with the same procedure. Flt3 receptor was inhibited using sunitinib (SU11248, Pfizer). Treatment was given by gavage (day 7–28, 40 mg/kg mouse), and the control group was treated with the same volume of citrate buffer as previously described [44]. Specificity of sunitinib for Flt3 inhibition in our model has been previously shown [44].

**Table 3:** Primers for real time PCR.

Target	Forward primer		Reverse primer	
***Survivin140***	TGGACAGACAGAGAGCCAAG	Exon 3	CTGACGGGTAGTCTTTGCAG	Exon4
***Survivin121***	GTCAAGAAGCAGATGGAAG	Exon 3	TCAGTCCTTATTCTCAATCAT	Retained part of intron3
***Survivin40***	CCTCAAGAACTACCGCATCG	Exon 1	TATGCTCCTCTTCGCTCTGG	Exon 1/3
***Total survivin***	AGATCTGGCAGCTGTACCTCA	Exon 1	AGTTCTTGAAGGTGGCGATG	Exon 1
***Survivin140*** ** + ** ***Survivin121***	TCGCCACCTTCAAGAACTG	Exon 1	ATCAGGCTCGTTCTCGGTAG	Exon 2

### Knockdown of Survivin using Lentiviral Vector

Lentiviral construct, encoding short hairpin (sh)RNA targeting survivin with the sequence CCGGCAAAGACTACCCGTCAGTCAACTCGAGTTGACTGACGGGTAGTCTTTGTTTTTG (TRCN0000054616, Sigma-Aldrich), were selected after *in vitro* evaluation. Lentiviral particles were given by a single intra-articular injection at immunization day 0 (10^7^ transduction particles/knee, n = 8). The control group received the same amount of lentiviral particles with non-targeting scrambled sequence (n = 9). Downregulation of survivin was confirmed in bone marrow and spleen.

### Cell Preparation and Cell Culture

Bone marrow cells from femur and tibia were flushed with PBS. Single cell suspensions from spleens were prepared by mechanically disruption of tissues through a 70 µm cell strainer. Erythrocytes were lysed in NH_4_Cl solution (0.83%, pH 7.29). Leukocyte pellet was resuspended in Iscoves complete medium (10% FBS, 4 mM L-glutamine, 50 µM β-mercaptoethanol, 50 µg/ml gentamycin sulphate ) or FACS buffer (PBS, 10% FBS, 0.09% NaN3, 0.5 mM EDTA). Freshly prepared mouse spleen cells were plated on 96-well plate at 2×10^5^ cells/well in Iscoves complete medium. Supernatants were collected after 48 hours. Cell lysates were prepared by freezing the cells in −20°C overnight in 6 M urea.

### Determination of Survivin and Flt3 Ligand Levels

Survivin and Flt3L protein levels in plasma, synovial fluid, supernatant and lysates were determined by ELISA (DYC647E, DY308, DY427, R&D Systems). Plasma and synovial fluid samples were tested in parallel ELISA plates, in dilution 1∶10, lysates 1∶3 and supernatant 1∶2 in PBS-BSA. The obtained absorbance values were compared to serial dilution of the recombinant proteins and presented as pg/mL. For intracellular staining of survivin, cells were permeabilized with Cytofix/Cytoperm™ Fixation/Permeabilization Solution Kit (BD Pharmingen™) and stained with anti-Survivin antibody (IC6472P, clone 91630, R&D Systems) or with isotype-matched IgG (IC002P, clone 11711, R&D systems).

### Flow Cytometry

2×10^6^ cells were plated on a 96-well plate, washed with FACS buffer and pelleted (4 min, 1200 rpm, 4°C). Cells were pre-incubated with Fc-block (BD Biosciences) for 30 minutes at 4°C and stained at 4°C for surface markers. Flt3 expression was detected using a biotinylated anti-mouse CD135 antibody (A2F10, Biolegend) followed by a Streptavidin-APC incubation (BD Biosciences). Then the following antibodies against surface markers were used; CD3-APC-eFluor® 780 (17A2), MHC-II-eFluor® 450 (M5/114.15.2), (eBioscience), Gr-1-PerCp (RB6-8C5), CD11b-PerCp (M1/70) (Biolegend), B220-FITC (RA3-6B2), CD11b-V450 (M1/70), CD11c-APC (HL3), B220-V500 (RA3-6B2) (BD Biosciences). Following staining, cells were washed, resuspended in FACS buffer, and collected in FACSCanto II equipped with FACSDiva software (BD Biosciences). Data were analyzed using the FlowJo software (Tree Star Inc., Ashland, OR). The gating of the cells was based on the isotype control values as well as fluorochrome minus one (FMO) settings when needed. Data is shown as percentage or geometric mean fluorescence intensity (gMFI), calculated in FlowJo and compared to data acquired on the same run.

### Protein Preparation and Western Blotting

Total protein was prepared from cells and tissue by homogenization and sonication in the presence of protease inhibitors (Complete Mini, Roche Diagnostics GmbH, Mannheim, Germany). Protein concentrations were measured using the BCA Protein Assay kit (Pierce, Rockford, IL) according to the manufacturer’s protocol. Proteins were separated on SDS-PAGE 4–12% Bis-Tris gels (NuPAGE, Invitrogen), and transferred to PVDF membranes (NuPAGE, Invitrogen). Membranes were blocked with 5% non-fat milk and incubated with rabbit-anti-survivin antibodies (840471, R&D systems) at 4°C over night. Detection was performed with peroxidase conjugated anti-rabbit secondary antibody (NA934VS, GE Healthcare) and ECL Prime™ Western Blotting Reagents (GE Healthcare). Chemiluminescent signals were visualized by the Chemidoc equipment and Quantity-One software (BioRad Laboratories).

### Gene Expression Analysis

Total RNA from spleen tissue and bone marrow cells was extracted using the RNeasy Mini Kit (Qiagen, Valencia, CA) according to the manufacturer’s instructions. Concentration and quality of the RNA was evaluated with NanoDrop spectrophotometer (Thermo Scientific, USA) and Experion (BioRad Laboratories). 400 ng RNA was used for cDNA synthesis using RT^2^ First Strand Kit (SABiosciences, Qiagen). Real-time amplification was performed with RT^2^ SYBR® Green qPCR Mastermix (SABioscences, Qiagen) using a ViiA™ 7 Real-Time PCR System (Applied Biosystems) according to the manufacturer’s instructions. A negative control (no template) reaction was also performed for each primer pair tested. A melting curve for each PCR was performed between 60 and 95°C to ensure that only a single product had been amplified. For *Flt3* and *Flt3L* PCR Assays from SABiosciences (Qiagen) were used (sequences available upon request). Primers used for quantification of *survivin* expression were designed using Primer3 [50], uniqueness checked using the In silico PCR feature of the UCSC Genome Browser, and NetPrimer (PremierBiosoft) was used to check for secondary structures. Primers were ordered from Sigma-Aldrich, and are noted in table 3. Expression levels of the genes were normalized to the two reference genes, *Gapdh* and *Ppia* (TATAA, Sweden, sequences available upon request). Primer concentration used was 0.5 µM. The results were expressed as the fold change compared with the expression level in the control cells with the ddCq-method.

### Statistical Analysis

Patient data was expressed as mean (±SD) and analyzed with GraphPad Prism, version 5.0 for Mac (GraphPad, San Diego, CA). The patient material was stratified by the level of survivin, and the difference between the groups regarding Flt3L levels, age, rheumatoid factor, disease duration, presence of erosions, CRP, and WBC count in blood and synovial fluid, was calculated using the Mann-Whitney test or Chi-Square test for categorical data. Experimental data was also analyzed using GraphPad Prism, expressed as mean with SD and significance regarding differences between groups was calculated using the Mann-Whitney test. The two-tailed tests were used and for the statistical evaluation of the results p values <0.05 was considered significant.
